# 

**Published:** 1887-02-05

**Authors:** 


### THE HOSPITAL

An Institution, Family, and Parochial Journal of
Hospitals, Asylums, & all Agencies for the Care of the Sick, Criticism & News.

### CONTENTS.

**Workers *v.* Beggars** - - - - - - - 311

**Words of Consolation.**—XIV. Nurses' Trials—Weariness - - - - - - - - 312

**"He Laid Down His Life."** An Infirmary Story. By C. Grant-Furley - - - - - 313

**A Noble Profession: Nursing the Sick.**—Nursing at Queen Charlotte's Lying-in Hospital. By Thomas Ryan, Secretary - - - - - - 315

**Ulcerated Legs: their Care and Cure.** By Mrs. Black, founder of St. Mary's Cottage Hospital, Southampton - - - - - - - - 316

**Workhouse Visiting and Management** - 317

**Notes and News.**—Items.—The Duke of Cambridge and the German Hospital.—The Metropolitan Convalescent Institution.—Hospital Saturday at New York.—Vacancies.—Hospital Sunday in the Metropolis.—Lord Chelmsford and the Convalescent Dinner Society.—The Royal Maternity Charity.—Medical Charities.—The Bristol Dispensary.—Overcrowding at Hospitals.—Fancy Bazaars in Aid of the Hospitals.—Memorial at Leicester Infirmary, etc., etc. - - 318

**Annotations.**—A Libel on Hospital Sunday.—The Home and the Hospital at Bristol - - - 321

**The Common Accidents of Every-day Life.** By A. W. Hare, M.B., F.R.C.S.E.—Early Misadventures: 1. Dangers Connected with Swallowing; 2. Dangers from Carelessness - - - - - - 322

**The Latest of the Water Cures** - - - 323

**Incidents of Animal Life.** Collated by the Rev. F. O. Morris, B.A. - - - - - - 324

**Amusement with Profit** - - - - - 234

**The Children's Corner.**—Puzzles, Answers, etc. - v

